# Compressive Coronary Intramural Hematoma: A Case Report

**DOI:** 10.7759/cureus.23283

**Published:** 2022-03-18

**Authors:** Achmad F Yahya, Aninka Saboe

**Affiliations:** 1 Department of Cardiology and Vascular Medicine, Faculty of Medicine, Universitas Padjadjaran, Dr. Hasan Sadikin General Hospital, Bandung, IDN; 2 Department of Cardiology and Vascular Medicine, Universitas Padjadjaran, Bandung, IDN

**Keywords:** cutting balloon, grenadoplasty, acute vessel closure, iatrogenic coronary artery dissection, intravascular ultrasound (ivus)

## Abstract

Despite the advancement of various devices and techniques, percutaneous coronary intervention (PCI) of chronic total occlusion (CTO) lesion remains one of the major challenges in interventional cardiology fields. It is also associated with higher complications than non-CTO-PCI due to procedural complexity. We presented a case of iatrogenic compressive coronary intramural hematoma (IMH) as CTO-PCI complications, which was judiciously detected and successfully managed with a cutting balloon.

## Introduction

Percutaneous coronary intervention (PCI) for chronic total occlusions (CTO) remains one of the most challenging lesion subsets in interventional cardiology. The development of technology and medical devices, advancement of PCI technique, and increasing operator expertise have improved efficacy and safety; however, complications are unavoidable due to its procedural complexity. When a complication occurs, prompt recognition is vital to avoid major sequelae [1]. Iatrogenic coronary intramural hematoma (IMH) is an uncommon complication that could occur during the PCI procedure. The incidence and natural history of IMH are not entirely understood, but several reports described various outcomes [2,3]. To date, there is no uniformity in IMH management; hence, management is currently made on a case-by-case basis, with no evidence-based guidelines to assist the operator. We present a case of CTO-PCI complication manifesting as coronary IMH, which is diagnosed with intravascular ultrasound (IVUS) and successfully managed with a cutting balloon.

## Case presentation

History of presentation

**Figure 1:**
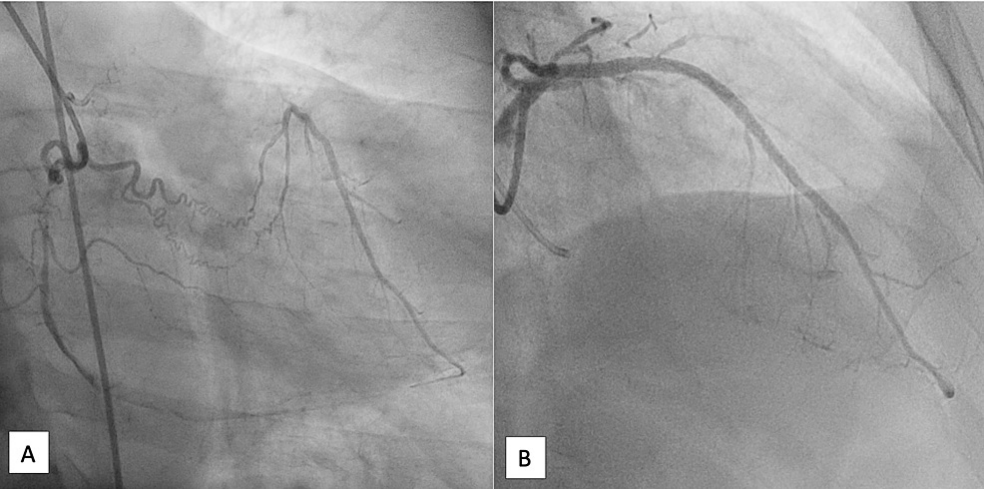
Angiogram (A) Initial angiogram revealed collateral flow to the LAD. (B) Final angiogram showed good results with TIMI 3 flow.

A 57-year-old male came to our outpatient department with a chief complaint of exertional dyspnea despite optimal heart failure therapy. His risk factors were hypertension and smoking. His echocardiogram revealed LVEF of 39%, with regional wall motion abnormalities at anterior and inferior segments. Dobutamine stress echocardiogram showed a large ischemic area at anterior and inferior segments, and a nitrate-augmented nuclear viability scan revealed that all left ventricular segments were viable. The patient was referred for catheterization, which revealed complex lesions: CTO at the LAD, CTO at the RCA, and significant stenosis at the LCx (Figure 1A).

He was suggested for CABG, but after a thorough discussion with the multidisciplinary heart team, he refused. PCI was performed one month later.

CTO-PCI LAD was performed with a dual angiography, antegrade approach. Runthrough NS Floppy Guidewire (Terumo, Tokyo, Japan) with Mogul (Nipro, Osaka, Japan) microcatheter as backup support was chosen in the first place; nonetheless, it was unsuccessful. Further attempts using Pilot 50 Guidewire (Abbott, USA) with Corsair microcatheter (Asahi, Aichi, Japan) successfully crossed the CTO lesion. Nonetheless, the wire could be advanced to the distal, and the microcatheter could not pass the lesion, as well as a 1.0/10 Sapphire II (OrbusNeich, Hongkong, China) semi-compliant balloon (SCB). Hence, we proceeded with grenadoplasty using 1.0/10 mm and 1.5/10 mm Sapphire II (OrbusNeich, Hongkong, China) SCB.

After grenadoplasty, the microcatheter finally could cross the lesion. Collateral contrast injection revealed reduced antegrade flow at the mid-distal LAD segment with an impression of dynamic compression (Video 1).

**Video 1:** Dynamic compression of the LAD Contralateral injection revealed reduced antegrade flow with suggestive dynamic compression. No radiolucent angiographic feature typical for dissection was observed.

Differential diagnosis

The reduced antegrade flow was suspected to have been caused by coronary artery dissection; however, the angiogram did not reveal a radiolucent pattern that is typical for dissection. Therefore, compressive intramural hematoma was suggested.

Management: PCI procedures

**Figure 2:**
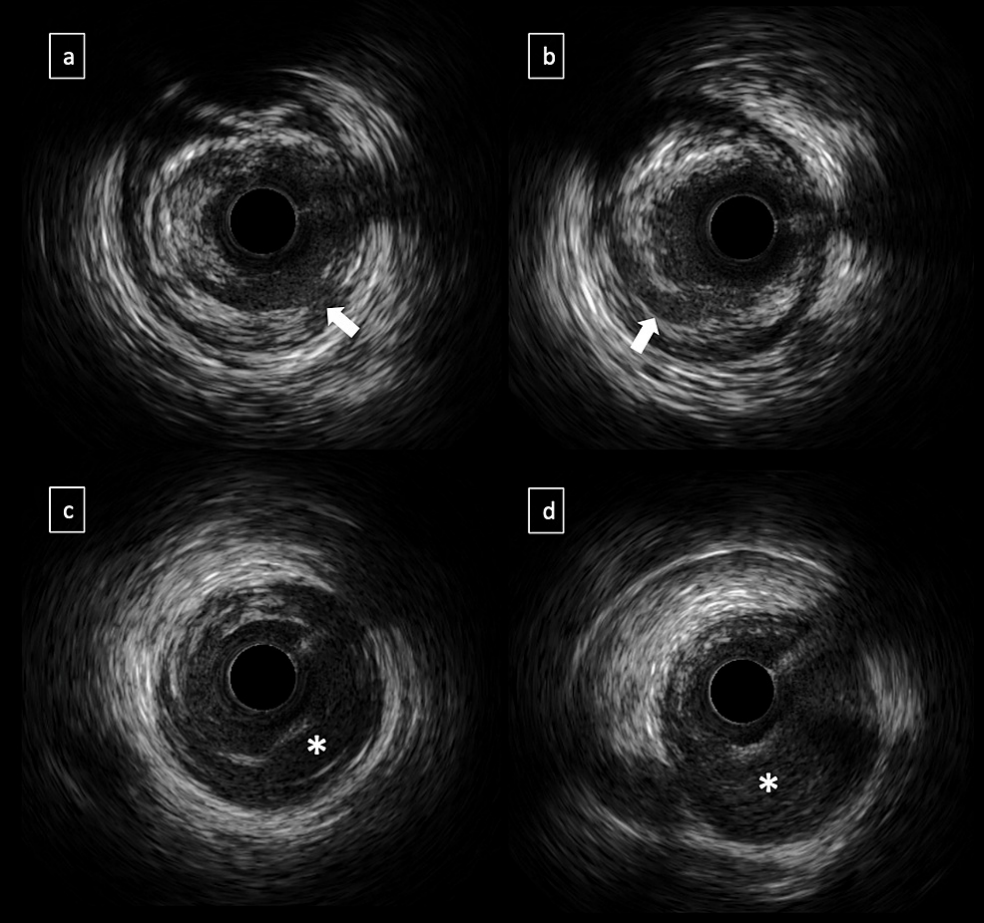
IVUS study (a and b) Intimal tear with flap (arrows) and (c and d) intramural hematoma (asterisks) compressing the true lumen.

Although the patient did not experience any symptoms and was hemodynamically stable, the angiogram revealed reduced antegrade flow at the mid-distal LAD segment with an impression of dynamic compression; hence, intracoronary imaging was performed to identify the etiology. Intravascular ultrasound (IVUS) evaluation revealed intimal tear with flap and intramural hematoma compressing the true lumen (Figure 2, Video 2).

**Video 2:** IVUS evaluation revealed an intramural hematoma The intramural hematoma was detected with intimal tear, clearly visualized flap, and lumen narrowing.

We performed focused-directed management to intramural hematoma using a 3.0/10 mm cutting balloon Wolverine (Boston Scientific, Marlborough, MA, USA) at the compromised segments, which inflated to nominal pressure. IVUS evaluation revealed reduced hematoma volume, while the dissected arterial wall and clear intimal tear were still detected (Video 3).

**Video 3:** IVUS evaluation after cutting balloon dilation The hematoma volume was reduced, while the dissected arterial wall and intimal tear were still detected.

To seal the intimal tear and the residual hematoma, a 3.0/38 mm Xience Xpedition LL drug-eluting stent (Abbott Vascular, Chicago, IL, USA) was deployed at mid-distal, followed by a 3.5/38 mm Xience Xpedition LL drug-eluting stent (Abbott Vascular, Chicago, IL, USA) placed at proximal mid-LAD.

Stent optimization at distal stent segment was performed with a 3.0/10 mm NC Sapphire Balloon (OrbusNeich, Hongkong, China), which inflated up to 18 atm.

Final IVUS evaluation revealed good stent expansion and apposition without stent edge dissection. Final angiogram showed good results with TIMI 3 flow (Figure 1B).

Follow-up

The patient was discharged on the following day and was asymptomatic at follow-up three months later.

## Discussion

Prior studies have described the clinical benefit of CTO-PCI. However, it is still associated with various complications that may significantly impact patient outcomes; hence, prompt recognition is essential to prevent unfavorable outcomes. In our case, we assumed that the complication was due to the grenadoplasty technique. This technique, also known as balloon-assisted microdissection, is a relatively safe plaque modification method for balloon uncrossable lesions by intentional balloon rupture [4]. However, the complications were detected early; hence, preventive measures for worsening conditions were performed.

In our case, although the patient did not experience any symptoms and was hemodynamically stable, we suspected that a complication occurred due to the angiographic appearance of antegrade flow restriction with dynamic compression of the vessel, which could be as an initial condition prior to acute vessel closure. One of the most common causes of acute vessel closure in PCI is coronary dissection, which could be detected from the radiolucent angiographic appearance during contrast injection [5]. Another possible cause is an intramural hematoma, in which angiographic findings include encroachment of the lumen and absence of a dissection flap, and there were no angiographic abnormalities in one-third case. Therefore, in those conditions, IVUS is useful to differentiate these conditions [6].

Our IVUS finding was consistent with intramural hematoma. Intramural hematoma is a rare PCI complication defined as localized blood accumulation within the media layer, with or without identifiable entry and exit points. IVUS detects an intramural hematoma by appearance or homogenous, hyperechoic, crescent-shaped area, which contains distinct echolucent zones within the hyperechoic regions [3].

Due to limited cases, there is no uniformity in the management of intramural hematoma; nevertheless, prior reports had described the possible role of cutting balloons in these cases [6-9]. The cutting balloon could create fenestration in the subintimal spaces, releasing the blood back into the lumen and relieving lumen compression. Vo et al. recommended using a balloon-to-artery ratio of 1:1 with nominal inflation pressure to minimize the risk of perforation [10]. As in our case, we chose the cutting balloon based on distal reference diameter and inflated it to the nominal pressure, which results in the restoration of antegrade coronary flow, leaving the dissection of which detected on the IVUS evaluation.

## Conclusions

Various recommendations had described the algorithm to approach coronary artery dissection and intramural hematoma, ranging from conservative to stenting strategy. In our cases, although the intramural hematoma had already resolved, the clearly visualized entry point without a clear exit point made a possible menace for recurring hematoma. Hence, we choose a stenting strategy and IVUS-guided PCI optimization with good final results.

## References

[1] Sutton NR, Bates ER (2019). Balancing the benefits, risks, and costs of chronic total occlusion percutaneous coronary intervention. Circ Cardiovasc Interv.

[2] Antonsen L, Thayssen P, Jensen LO (2015). Large coronary intramural hematomas: a case series and focused literature review. Cardiovasc Revasc Med.

[3] Maehara A, Mintz GS, Bui AB (2002). Incidence, morphology, angiographic findings, and outcomes of intramural hematomas after percutaneous coronary interventions: an intravascular ultrasound study. Circulation.

[4] Ye Y, Zhao X, Du J, Zeng Y (2020). Efficacy and safety of balloon-assisted microdissection with Sapphire® II 1.0-mm balloon in balloon-uncrossable chronic total occlusion lesions. J Int Med Res.

[5] Rogers JH, Lasala JM (2004). Coronary artery dissection and perforation complicating percutaneous coronary intervention. J Invasive Cardiol.

[6] Noh HJ, Choi JH, Song YB (2009). Intravascular ultrasound-guided troubleshooting in a large hematoma treated with fenestration using a cutting balloon. Korean Circ J.

[7] Miura Y, Koyama K, Kongoji K, Soejima K (2021). Fenestration using a novel cutting balloon for acute vessel occlusion secondary to intramural hematoma following stent implantation. Cardiovasc Revasc Med.

[8] Koji S, Takeaki K, Hironao I (2017). TCTAP C-141 usefulness of releasing the intramural hematoma by cutting balloon for the wire-induced coronary artery dissection. J Am Coll Cardiol.

[9] Servoz C, Monségu J, Abdellaoui M, Faurie B (2021). Cutting balloon to treat post-stenting intramural hematoma during ST elevation myocardial infarction. Postepy Kardiol Interwencyjnej.

[10] Vo MN, Brilakis ES, Grantham JA (2018). Novel use of cutting balloon to treat subintimal hematomas during chronic total occlusion interventions. Catheter Cardiovasc Interv.

